# Defective but tumorigenic: the evolutionary and functional roles of mutated oncoviruses

**DOI:** 10.1093/femsre/fuaf048

**Published:** 2025-09-26

**Authors:** Yoshitaka Sato, Yusuke Okuno, Takayuki Murata, Hiroshi Kimura

**Affiliations:** Department of Virology, Nagoya University Graduate School of Medicine, Nagoya 466-8550, Japan; Department of Virology, Nagoya City University Graduate School of Medical Sciences, Nagoya 467-8601, Japan; Department of Virology, Fujita Health University School of Medicine, Toyoake 470-1192, Japan; Department of Virology, Nagoya University Graduate School of Medicine, Nagoya 466-8550, Japan

**Keywords:** human oncoviruses, defective viral genomes, integration, loss-of-function mutations, immune evasion, virus-host interactions

## Abstract

Human oncogenic viruses contribute significantly to the global health burden and include seven types: Epstein–Barr virus, hepatitis B virus, human T-cell leukemia virus type 1, human papillomavirus, hepatitis C virus, Kaposi’s sarcoma-associated herpesvirus, and Merkel cell polyomavirus. While the roles of latent or integrated viral genomes in cancer have been documented, emerging evidence highlights the contribution of defective viruses—those carrying intragenic deletions or loss-of-function mutations—in promoting viral oncogenesis. These altered genomes often lack genes essential for lytic replication or immune recognition, which enhances their persistence and immune evasion. In virus-associated diseases, specific patterns of gene retention and deletion suggest that host-driven selective pressures drive the emergence of these altered genomes. This review examines the generation, prevalence, and functional impact of these viruses, reframing them as active participants in disease development and progression. Recognizing their role offers new insights into viral tumor evolution and creates opportunities for applications in viral diagnostics and targeted intervention strategies.

## Introduction

Tumor development is shaped by multiple factors, including host genetics, environmental influences, immune status, aging, and pathogens. Among these, infection is a significant contributor, with viruses accounting for approximately 15% of all human cancers (Parkin 2006, Elbasir et al. 2023, Moore and Chang 2024). Since the discovery of the first human oncovirus more than 60 years ago (Epstein et al. 1964), substantial research has shed light on various viral oncogenic mechanisms. Nevertheless, a full understanding of how persistent viral infections lead to cancer remains elusive.

Defective viruses—those carrying partial genome deletions and loss-of-function mutations—are frequently found in tumor tissues (Moore and Chang 2010). Growing evidence suggests that these altered genomes are not simply inert byproducts of viral replication but are positively selected during tumor progression, representing viral adaptations to the neoplastic microenvironment. As such, defective viral genomes may play active, functional roles in supporting or even driving oncogenesis.

The generation and significance of defective viral genomes have been extensively studied in RNA viruses, which possess low-fidelity polymerases and are prone to frequent deletions (Genoyer and López 2019, Vignuzzi and López 2019). These defective genomes have been shown to aid in host cell adaptation, immune evasion, and persistent infection (Brennan and Sun 2024). By contrast, relatively less is known about the origins and functional consequences of defective genomes in DNA viruses, which typically exhibit lower mutation rates. Their broader roles in tumor progression across diverse human tumor viruses also remain incompletely understood.

This review synthesizes emerging evidence across human oncoviruses, focusing on the generation, prevalence, and biological impact of defective viral genomes. By highlighting both shared and virus-specific mechanisms, we aimed to reposition these genomes from incidental remnants to active participants in cancer evolution and to explore their emerging relevance as diagnostic or therapeutic targets.

## Overview of human oncoviruses

An oncovirus is defined as any virus that contributes to cancer development by promoting cell transformation and uncontrolled proliferation (Toner et al. 2024, Xiao et al. 2025). Viruses that induce tumors indirectly by weakening the immune system—such as human immunodeficiency virus type 1—are excluded from this category. There are seven known oncoviruses that affect humans: Epstein–Barr virus (EBV), hepatitis B virus (HBV), human T-cell leukemia virus type 1 (HTLV-1), human papillomavirus (HPV), hepatitis C virus (HCV), Kaposi’s sarcoma-associated herpesvirus (KSHV), and Merkel cell polyomavirus (MCPyV), listed in the order of their discovery (Epstein et al. 1964, Dane et al. 1970, Poiesz et al. 1980, Durst et al. 1983, Choo et al. 1989, Chang et al. 1994, Feng et al. 2008) (Table 1). Human oncoviruses include both DNA and RNA viruses with diverse classifications.

**Table 1.** Characteristics of 7 human oncoviruses.

Virus	EBV	HBV	HTLV-1	HPV	HCV	KSHV	MCPyV
Discovery year	1964	1970	1980	1983	1989	1994	2008
Genome (Size)	DNA (172 kb)	DNA (3.2 kb)	RNA (9.0 kb)	DNA (8.0 kb)	RNA (9.6 kb)	DNA (165 kb)	DNA (5.4 kb)
Family	Orthoherpesviridae	Hepadnaviridae	Retroviridae	Papillomaviridae	Flaviviridae	Orthoherpesviridae	Polyomaviridae
Main target cells	B cells, epithelial cells	hepatocytes. cholangiocytes	CD4^+^ T cells	basal cells of squamous epithelium	hepatocytes, lymphocytes	B cells, vascular endothelial cells	dermal fibroblasts
Viral oncoproteins	LMP1, -2, EBNA2, -3A, -3C	HBx	Tax, HBZ	E5, E6, E7	Core protein, NS3, NS5A	LANA, vCyclin, vFLIP, vIL-6, vGPCR	LT, ST
Targeted signaling pathways/molecules	NF-κ B, JAK/STAT, PI3K-AKT-mTOR, MAPK, WNT/β-catenin, c-Myc	p53, JAK/STAT, MAPK, Notch, WNT/β-catenin	PI3K-AKT-mTOR, NF-κB, JAK/STAT	p53, pRB, PI3K-AKT-mTOR, Notch	p53, NF-κB	p53, pRB, NF-κB, JAK/STAT, PI3K-AKT-mTOR, MAPK, Notch, WNT/β-catenin	pRB, PI3K-AKT-mTOR
Neoplastic diseases	Burkitt lymphoma, diffuse large B-cell lymphoma, extranodal NK/T-cell lymphoma, chronic active EBV disease, nasopharyngeal carcinoma, gastric carcinoma	hepatocellular carcinoma, intrahepatic cholangiocarcinoma	adult T-cell leukemia/lymphoma	cervical cancer, anal cancer, oropharyngeal cancer, vulvar and vaginal cancers, penile cancer	hepatocellular carcinoma, marginal zone lymphoma	Kaposi’s sarcoma, primary effusion lymphoma, multicentric Castleman disease	Merkel cell carcinoma
% of cancer*	1.0	3.1	0.03	5.2	1.8	0.9	<0.1

Abbreviations: EBV, Epstein–Barr virus; HBV, hepatitis B virus; HTLV-1, human T-cell leukemia virus type 1; HPV, human papillomavirus; HCV, hepatitis C virus; KSHV, Kaposi’s sarcoma-associated herpesvirus; MCPyV, Merkel cell polyomavirus; LMP, latent membrane protein; EBNA, EBV nuclear antigen; HBx, HBV X protein; HBZ, HTLV-1 basic leucine zipper factor; LANA, latency-associated nuclear antigen; LT, large T antigen; ST, small T antigen.

* Estimates based on Parkin DM, 2006.

The mechanisms by which human oncoviruses induce tumor formation vary depending on the virus and tumor type (Mesri et al. 2014, Krump and You 2018, Xiao et al. 2025). Nevertheless, the tumorigenesis of infected cells is generally thought to follow a multi-stage oncogenesis model, similar to that of other cancers (Barcellos-Hoff et al. 2013, Kandoth et al. 2013). In many cases, viral infection contributes to early tumorigenic processes; however, it may also occur in cells that already harbor genetic mutations or epigenetic modifications. External factors such as environmental exposures and inflammation further promote genetic damage in host cells. Over time, these cells accumulate genetic abnormalities and epigenetic modifications, enabling them to acquire properties such as immortalization, invasiveness, and metastatic potential—ultimately leading to malignant transformation. The viral infection is believed to inactivate tumor suppressor proteins such as p53 and pRB and/or activate intracellular signaling pathways, including PI3K-AKT-mTOR, NF-κB, and JAK/STAT. These changes, mediated by viral oncoproteins, drive neoplastic progression (Mesri et al. 2014, Krump and You 2018, Xiao et al. 2025) (Table 1). In addition, the mutational landscapes of virus-associated and non-associated cancers at the same anatomical site can differ, as seen in EBV-positive versus EBV-negative gastric carcinomas. EBV-positive gastric carcinomas are characterized by frequent PIK3CA mutations, DNA hypermethylation, and JAK2/PD-L1/PD-L2 amplifications, which distinguish them from EBV-negative cases that more commonly harbor p53 mutations (Cancer Genome Atlas Research Network 2014).

## Defective viral genomes: concepts and emerging importance

Oncogenesis is not inevitable for viruses and does not necessarily confer an evolutionary advantage (Moore and Chang 2024). In fact, malignant transformation is often detrimental to viruses because it can compromise host survival. Moreover, human lifespans have only begun to extend significantly in recent history, and most cancers develop in older individuals; this suggests that oncogenesis was not originally embedded in the viral life cycle. Instead, it is plausible that certain viral defects—especially those impairing immune recognition or enhancing persistence—confer a selective advantage to infected cells within the tumor environment, allowing them to evolve and survive. Recurrent deletions in the BamHI-A rightward transcripts (BARTs) region of EBV, the HBV X protein (HBx), the HTLV-1 transactivator protein Tax, the E2 gene of HPV, and the truncated large T antigen (LT) of MCPyV support the idea that such alterations are positively selected and functionally relevant in oncogenesis (Takeda et al. 2004, Sung et al. 2012, Burk et al. 2017, Harms et al. 2018, Okuno et al. 2019) (Table 2). These are not random byproducts of replication failure but appear to contribute actively to tumor persistence and progression. By lacking genes essential for lytic replication and immune recognition, defective viruses facilitate immune evasion and long-term latency. This selection may be further reinforced by the tumor microenvironment, where impaired antigen presentation and local immunosuppression favor the clonal expansion of cells harboring defective viruses. In this ecological niche, such cells can evade immune clearance and persist within tumors, thereby accelerating neoplastic progression (Galassi et al. 2024).

**Table 2.** Contribution of defective viruses to tumor formation.

Virus	EBV	HBV	HTLV-1	HPV	HCV	KSHV	MCPyV
Associated neoplasms	diffuse large B-cell lymphoma, extranodal NK/T-cell lymphoma, chronic active EBV disease	hepatocellular carcinoma	adult T-cell leukemia/lymphoma	cervical cancer	hepatocellular carcinoma	Kaposi′s sarcoma	Merkel cell carcinoma
Affected genes/molecules	BART miRNA, EBNA3B, BALF genes, C promotor	HBx C-terminal, Pre-S/S	Tax exon 2, 5′ LTR	E2	unknown	K8.1 K5-K6	LT C-terminal
Mechanism	loss of BART miRNAs or BALF genes activates BZLF1, promoting proliferation, immune evasion, and host genome instability	HBx C-terminal mutants induce reprogramming of glucose metabolism; PreS/S mutants induce ER stress and genomic instability	loss of Tax expression promotes immune evasion	absence of E2 disrupts repression of E6/E7, facilitating oncogenesis	none	K8.1 loss facilitates escape from cellular and humoral immune responses	LT truncation disables viral replication but enhances tumor growth and survival
Integration (frequency)	no (occasional), maintained as episomes	yes (60%–90% in hepatocellular carcinoma)	yes (100%)	yes (70%–100% in cervical cancer)	no	no, maintained as episomes	yes (100% in MCPyV + Merkel cell carcinoma)
Contribution	moderate	high	High	high	exceptionally low if any	moderate	extremely high

Abbreviations: EBV, Epstein–Barr virus; HBV, hepatitis B virus; HTLV-1, human T-cell leukemia virus type 1; HPV, human papillomavirus; HCV, hepatitis C virus; KSHV, Kaposi’s sarcoma-associated herpesvirus; MCPyV, Merkel cell polyomavirus; BART miRNA, *Bam*HI-A rightward transcripts micro RNA; EBNA, EBV nuclear antigen; BALF, *Bam*HI-A leftward open reading frame; HBx, HBV X protein; LTR, long terminal repeat; LT, large T antigen; ER, endoplasmic reticulum.

**Figure 1.**
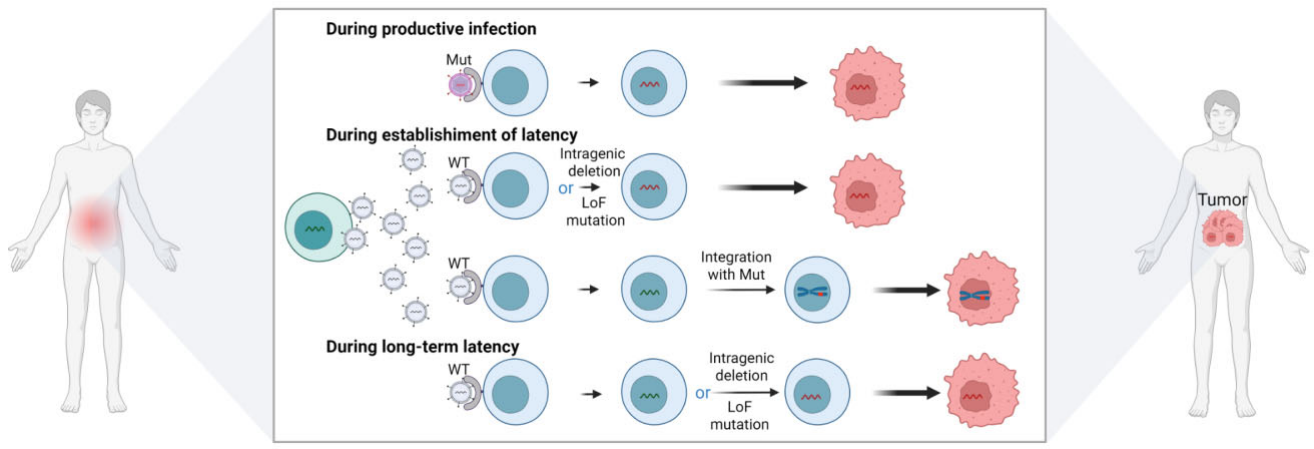
Conceptual framework for the evolution of defective viral genomes in virus-associated tumorigenesis. Defective viral genomes can arise during productive infection, establishment of latency, or long-term latency, and may promote tumorigenesis via persistence, integration, or functional mutations. Mut, mutated virus; WT, wild-type virus; LoF, loss-of-function. Created in https://BioRender.com

Many defective viruses have lost their productive infectivity and have limited ability to spread between cells or individuals. Instead, they remain within infected cells and are passed on to progeny cells as those cells divide. There are two main forms of defective virus: intragenic deletions and point or small-scale mutations (such as single-nucleotide changes or small insertions/deletions) that result in loss of function. Although the detection of intragenic deletions has improved with advances in next-generation sequencing, it is likely that defects caused by single-nucleotide mutations are often overlooked—partly because the functional domains of many viral genes remain poorly characterized. This is particularly evident in viruses with large genomes, such as EBV and KSHV, which encode more than 80 genes (Kanda et al. 2019). These defects can arise at various stages: during productive infection, during the establishment of latency—either as episomes or, in some cases, via chromosomal integration—and even during long-term maintenance of latency (Fig. 1). An additional, not mutually exclusive, possibility is that tumorigenesis itself precedes viral genome alteration, with the highly unstable genomic environment characteristic of cancer cells subsequently giving rise to defective viral genomes.

Four viruses—HBV, HTLV-1, HPV, and MCPyV—are often or consistently integrated into host chromosomes (Vojtechova and Tachezy 2025) (Table 2). By contrast, two viruses—EBV and KSHV, both members of the Orthoherpesviridae family—do not normally integrate into host DNA but, instead, remain latent in the nucleus as episomes, which are passed on to daughter cells during division. Among the seven human oncoviruses, the involvement of defective viruses in HCV appears limited or indirect. This is primarily because chronic inflammation is the main driver of HCV-related carcinogenesis, and the virus neither integrates into host chromosomes nor maintains long-term episomal latency (Krump and You 2018, Xiao et al. 2025). In the next chapter, we will focus on six oncoviruses excluding HCV and outline the relationship between the emergence of defective viruses and tumor promotion in each of them.

## Defective genomes in individual tumor viruses

### EBV and lymphoma

**Figure 2.**
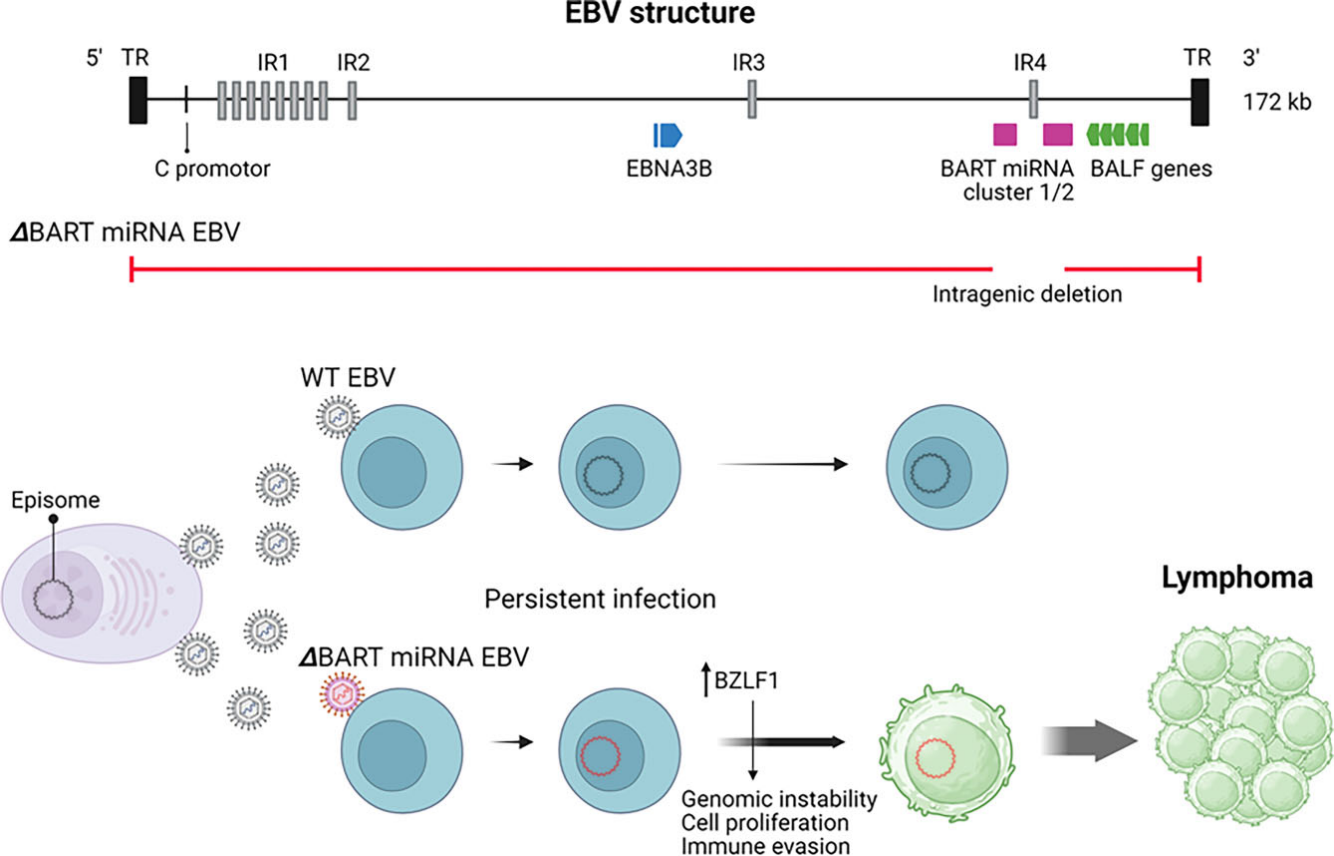
Epstein–Barr virus (EBV) and lymphoma. Intragenic deletions occur in *Bam*HI-A rightward transcripts microRNA (BART miRNA) clusters, EBV nuclear antigen 3B (EBNA3B), C promoter, and *Bam*HI-A leftward open reading frame (BALF) genes. Loss of BART miRNAs activates BZLF1, leading to host genome instability, cell proliferation, and immune evasion. TR, terminal repeat; IR, internal repeat. Created in https://BioRender.com.

EBV, a double-stranded DNA virus belonging to the Orthoherpesviridae family, is associated not only with various lymphoid malignancies—including Burkitt lymphoma, diffuse large B-cell lymphoma, and extranodal NK/T-cell lymphoma—but also with epithelial tumors such as nasopharyngeal and gastric carcinomas (Alaggio et al. 2022, Campo et al. 2022, Gewurz et al. 2022, Munz 2025). Following primary infection, EBV establishes latency by persisting as an episomal DNA element within the nucleus of host cells (Fig. 2). A very recent comprehensive genetic analysis of 990 EBV strains revealed that a wide range of viral genes are defective in tumors, with defective viruses particularly concentrated in lymphoid malignancies (Khine et al. 2025). It should be emphasized, however, that defective genomes are not invariably present in all EBV-associated tumors. Notably, they are observed more frequently in lymphoid than in epithelial malignancies. One possible explanation is that cytidine deaminases such as APOBEC3 and activation-induced cytidine deaminase, which are highly expressed in hematopoietic cells, introduce DNA mutations and strand breaks that destabilize the viral episome (Summerauer et al. 2022). This process may be further modulated by viral countermeasures such as BORF2, which antagonizes APOBEC3B to maintain genome integrity; its loss has been shown to cause extensive hypermutation (Cheng et al. 2019). Together, these cellular and viral factors may help explain the tissue specificity of defective EBV genomes. In addition, most deletions in EBV are thought to arise during viral production, with terminal and internal repeats providing a substrate for recombination, although other mechanisms may also contribute. By contrast, the frequent C-to-T substitutions in EBV genomes are more likely to accumulate while the virus persists as an episome during latency (Khine et al. 2025). These arise through deamination of methylated cytosines followed by incomplete repair, a process that parallels mutational mechanisms in the human genome.

The most frequently affected regions are the BART microRNA (miRNA) cluster, followed by EBV nuclear antigen 3B (EBNA3B), the C promoter, and BamHI-A leftward open reading frame (BALF) genes, which are involved in viral replication (Okuno et al. 2019, Peng et al. 2019, Mabuchi et al. 2021, Venturini et al. 2021, Wongwiwat et al. 2022, Ito et al. 2024, Khine et al. 2025). The mechanisms by which these gene defects promote tumorigenesis vary, and the frequency of defects differs across lymphoma subtypes: Burkitt lymphoma (25%), extranodal NK/T-cell lymphoma (41%), chronic active EBV disease (28%), and EBV-positive diffuse large B-cell lymphoma (48%) (Khine et al. 2025).

EBV encodes more than 40 miRNAs, including two major clusters in the BART region. Among these, ebv-mir-BART6, ebv-mir-BART18, and ebv-mir-BART20 negatively regulate the EBV transactivators BZLF1 and BRLF1 (Iizasa et al. 2020, Kimura et al. 2021). Mutant EBV strains lacking these regions have been shown to enhance lymphomagenesis through BZLF1 induction in xenograft models (Lin et al. 2015). Deletion of BART miRNAs induces expression of lytic infection-related genes triggered by BZLF1. Several of these lytic genes—such as BNRF1, BGLF5, and BALF3—participate in destabilizing the host genome, while BHRF1 (a viral BCL-2 homolog) and BCRF1 (a viral interleukin-10 homolog) promote cell proliferation. Thus, in cells lacking portions of the BART miRNA region, BZLF1 suppression is lifted, activating downstream genes believed to drive cell proliferation, immune evasion, and host genome instability (Münz 2019, Murata et al. 2021) (Fig. 2). Similarly, deficiencies in the BALF gene group—which includes EBV lytic replication genes—also promote tumorigenesis by increasing BZLF1 expression (Okuno et al. 2019, Khine et al. 2025). Because deletion of the BART miRNAs or the BALF gene group completely or partially impairs the production of progeny viruses, these defective viruses are unlikely to spread within the host or between individuals. Instead, cells infected with such defective viruses persist through positive selection and proliferate via host cell division.

By contrast, EBNA3B appears to promote tumorigenesis through a distinct mechanism. A prior study using mouse xenograft models demonstrated that EBNA3B deficiency enhances tumor formation, suggesting that this gene may function as a viral tumor suppressor (White et al. 2012). EBNA3B activates the human tumor suppressor genes PTEN and RB1, further supporting its role in limiting oncogenesis; loss of this function is associated with increased lymphomagenesis (Khine et al. 2025). EBNA3B deficiency can arise not only from large deletions but also from loss-of-function mutations, with specific mutational hotspots identified.

### HBV and hepatocellular carcinoma

**Figure 3.**
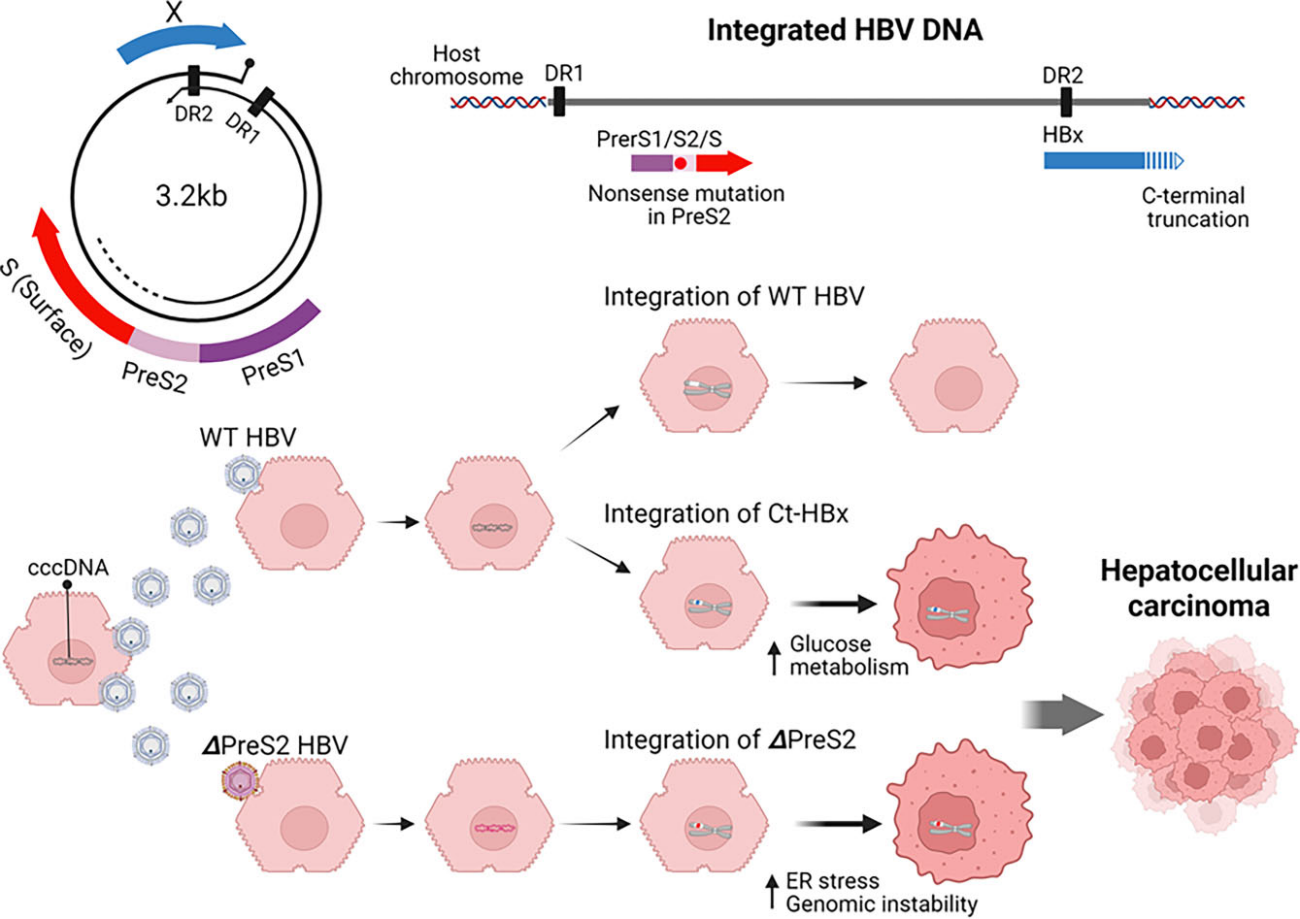
Hepatitis B virus (HBV) and hepatocellular carcinoma. C-terminal-truncated HBV X protein (Ct-HBx), generated upon viral DNA integration, induces reprogramming of glucose metabolism. PreS/S mutants, produced during virus replication, cause endoplasmic reticulum (ER) stress and genomic instability. DR, direct repeat; cccDNA, covalently closed circular DNA. Created in https://BioRender.com.

HBV is a partially double-stranded relaxed circular DNA virus that infects the liver and is strongly associated with chronic hepatitis, liver cirrhosis, and hepatocellular carcinoma (Levrero and Zucman-Rossi 2016). Upon infecting hepatocytes, HBV is converted into covalently closed circular DNA (cccDNA) in the nucleus (Fig. 3). This cccDNA serves as a template for transcribing pre-genomic RNA and synthesizing progeny DNA via reverse transcriptase. In hepatocytes affected by chronic hepatitis, HBV predominantly persists in the nucleus as cccDNA. However, in hepatocellular carcinoma tissue, integration of HBV into host chromosomes is observed in 86.4% of cancerous tissues and 30.7% of non-cancerous tissues (Sung et al. 2012). This integration occurs via linear double-stranded DNA—a byproduct of the viral replication process—rather than through cccDNA (Zoulim et al. 2024, Vojtechova and Tachezy 2025). The HBx gene, located at the 3′ end of the double-stranded genome, is often integrated in a form with a truncated C-terminus (Ct-HBx) (Sung et al. 2012).

HBx is an oncogene that activates the JAK/STAT, MAPK, Notch, and WNT/β-catenin pathways, promoting cell proliferation, invasion, metastasis, and immune evasion (Krump and You 2018, Xiao et al. 2025). Additionally, HBx inhibits p53, thereby suppressing apoptosis and promoting abnormal cell growth. Interestingly, the C-terminal region of HBx contains nuclear localization signals and inhibitory domains, and its deletion may partially reduce its transcriptional regulatory activity (Zoulim et al. 2024). Nonetheless, Ct-HBx has been shown to selectively retain—or even enhance—certain tumorigenic functions, including anti-apoptotic activity, metastasis promotion, and metabolic regulation. In fact, Ct-HBx loses its ability to induce apoptosis and, instead, strongly promotes hepatocyte proliferation (Ng et al. 2016). It has also been reported that Ct-HBx contributes to reprogramming glucose metabolism through TXNIP inhibition, thereby facilitating the development of hepatocellular carcinoma (Zhang et al. 2021). Thus, although Ct-HBx likely arises by chance during integration, its tumor-promoting properties appear to be positively selected and become dominant in hepatoma cells (Fig. 3).

In addition, various mutations are frequently observed in the PreS/S region in cases of hepatocellular carcinoma (Wang et al. 2006, Pollicino et al. 2014). Unlike HBx deletions, these mutations occur during viral replication. Because of the lack of proofreading activity in HBV reverse transcriptase, point mutations, deletions, and insertions arise naturally at high frequency. The PreS/S region, rich in repetitive sequences and recombination hotspots, is structurally unstable and particularly prone to accumulating mutations. Common mutations in this region include PreS1 deletions, PreS2 start codon mutations, partial PreS2 deletions, and truncations in the S region (Pollicino et al. 2014). Because the PreS/S region contains immunogenic epitopes for the HBs antigen, mutations here are likely selected for their advantage in immune evasion, particularly under pressure from anti-HBs antibodies. In the case of PreS2 deletion mutants, the resulting surface antigens accumulate in the endoplasmic reticulum (ER), leading to ER stress, DNA damage, and genomic instability (Liu et al. 2022) (Fig. 3). This chronic cellular damage and inflammation are strongly associated with the pathogenesis of hepatocellular carcinoma.

### HTLV-1 and adult T-cell leukemia/lymphoma

**Figure 4.**
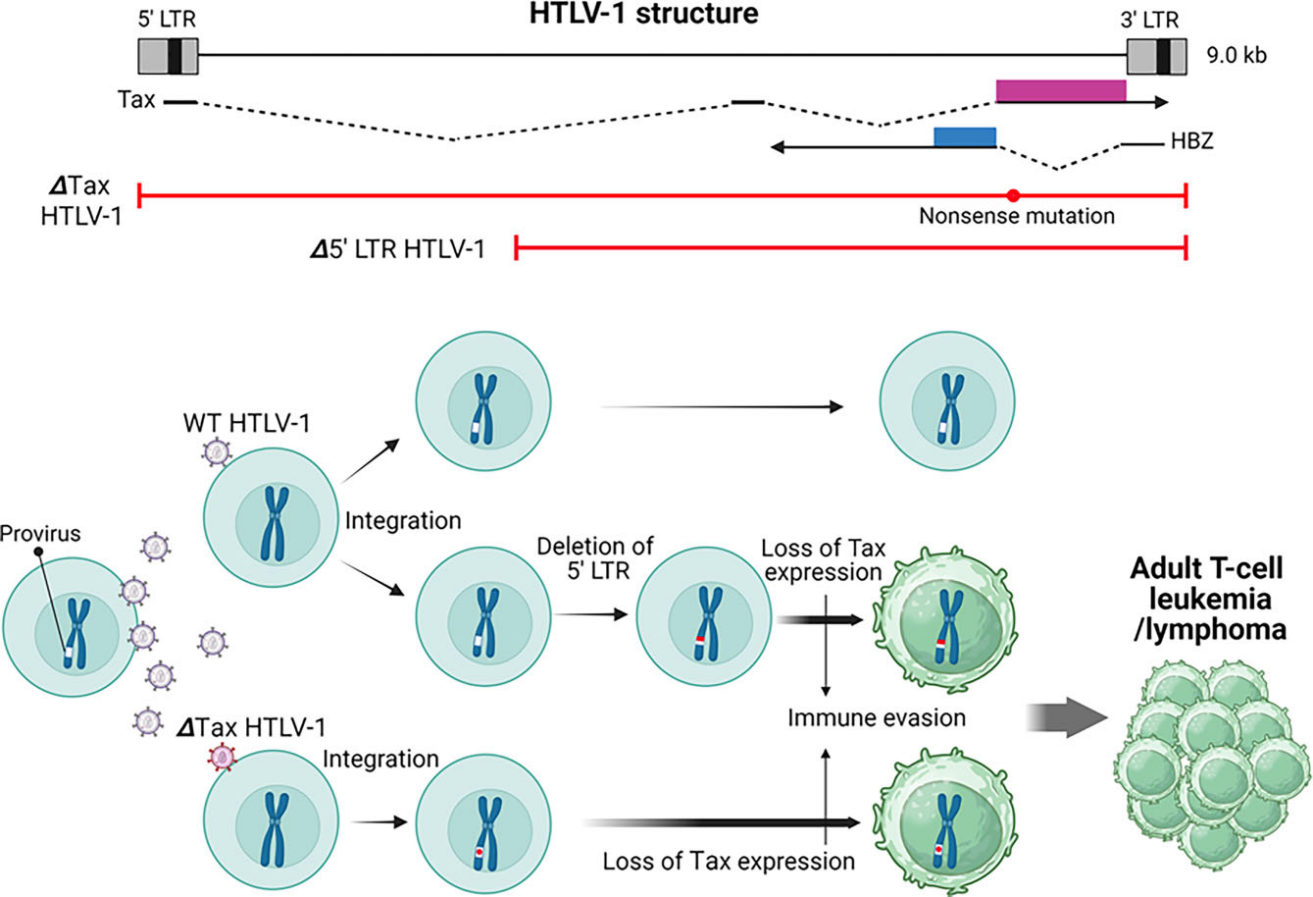
Human T-cell leukemia virus type 1 (HTLV-1) and adult T-cell leukemia/lymphoma. Loss of Tax expression, resulting from deletion of the 5′ long terminal repeat (LTR) or nonsense mutations in the Tax exon, promotes immune evasion. HBZ, HTLV-1 basic leucine zipper factor. Created in https://BioRender.com.

HTLV-1 is a retrovirus with a single-stranded RNA genome that causes adult T-cell leukemia/lymphoma (ATLL), an aggressive malignancy of CD4+ T lymphocytes (Bangham et al. 2019, Alaggio et al. 2022, Campo et al. 2022). It is primarily transmitted from mother to child through breastfeeding. After infection, HTLV-1 is reverse-transcribed into double-stranded DNA by viral reverse transcriptase and then integrated into the chromosomes of host CD4+ T cells as a provirus by the viral integrase (Fig. 4).

HTLV-1 encodes two major oncoproteins: Tax and HTLV-1 basic leucine zipper factor (HBZ). Tax activates the NF-κB and PI3K–Akt–mTOR pathways, promoting cell proliferation (Krump and You 2018), and plays a particularly critical role during the early stages of tumorigenesis (Bangham et al. 2019). However, Tax is rarely transcribed in ATLL tumor tissues (Takeda et al. 2004, Kataoka et al. 2015). The mechanisms of Tax inactivation fall into three categories: deletion of the 5′ long terminal repeat (LTR) promoter region (i.e. structural deletion of the provirus), epigenetic suppression through DNA methylation of the 5′ LTR, and mutations in the Tax gene itself (including missense or nonsense mutations, frameshifts, or complete deletions) (Kogure and Kataoka 2017, Bangham et al. 2019). The Tax protein is highly immunogenic and readily recognized by cytotoxic T lymphocytes, so HTLV-1–infected cells expressing Tax are efficiently eliminated by the host immune system. Consequently, tax-deficient or non-expressing cells are positively selected under immune pressure and undergo clonal proliferation (Fig. 4).

There is ongoing debate regarding when Tax inactivation occurs—whether during or after viral integration into host DNA (Bangham et al. 2019). Tax gene mutations in ATLL cells frequently show G-to-A transitions, a characteristic mutation pattern induced by APOBEC3G. Because APOBEC3G acts during reverse transcription, these mutations are thought to occur at the time of initial infection (Bangham et al. 2019). By contrast, deletions involving the 5′ LTR are generally considered to arise during clonal expansion and under immune selection pressure after integration, although the possibility that they occur earlier cannot be excluded. Notably, 5′ LTR deletions have also been detected in the peripheral blood of asymptomatic carriers (Katsuya et al. 2019). Moreover, 6-bp repeat sequences—believed to be a signature of viral integrase activity—are occasionally found in conjunction with 5′ LTR defects, supporting the idea that some of these alterations occur during integration (Miyazaki et al. 2007, Katsuya et al. 2019).

HBZ is the only viral gene consistently expressed in HTLV-1-infected cells. It promotes cell proliferation by driving the T-cell cycle and enhancing resistance to apoptosis (Bangham et al. 2019, Matsuoka and Mesnard 2020). Unlike Tax, HBZ is less immunogenic and is therefore less likely to be targeted by cytotoxic T lymphocytes. It also promotes the production of anti-inflammatory cytokines, such as IL-10, aiding immune evasion (Matsuoka and Mesnard 2020, Xiao et al. 2025). While Tax and other viral genes are transcribed from the 5′ LTR, HBZ is uniquely transcribed from the inverted 3′ LTR, allowing its expression to persist even when the 5′ LTR is deleted (Fig. 4).

### HPV and cervical cancer

**Figure 5.**
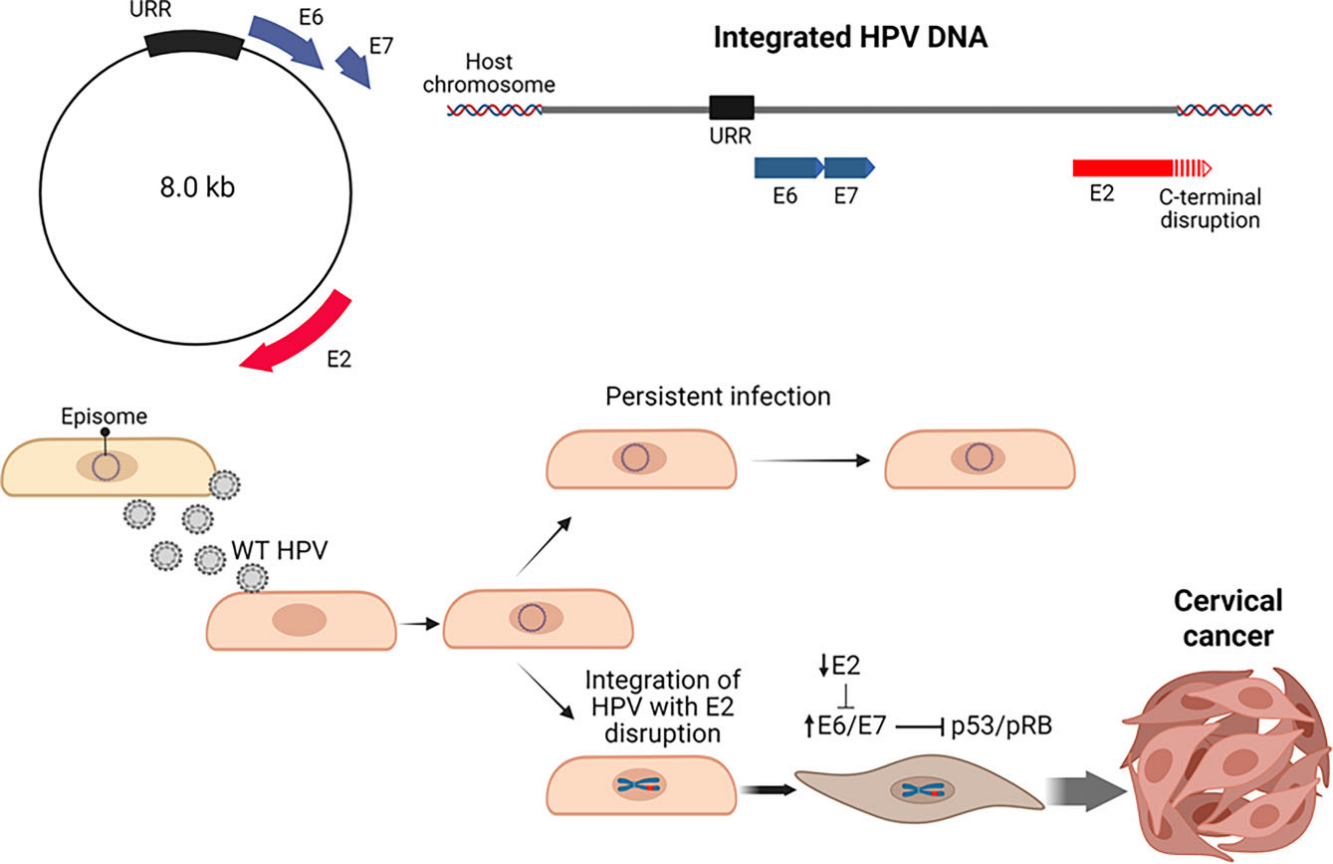
Human papillomavirus (HPV) and cervical cancer. Disruption of E2 following viral DNA integration leads to activation of E6/E7, thereby facilitating oncogenesis. URR, upstream regulatory region. Created in https://BioRender.com.

HPV is a circular double-stranded DNA virus that causes benign warts as well as cervical and other mucosal cancers (Schiller and Lowry 2022). High-risk types, such as HPV16 and HPV18, are strongly associated with cancer development. After infecting host basal cells in the cervical epithelium, HPV is typically maintained in episomal form (Zhang et al. 2025) (Fig. 5). However, as infected cells progress toward cervical cancer, the HPV genome is often integrated into the host genome. This integration is generally absent in asymptomatic individuals and in patients with low-grade cervical intraepithelial neoplasia, but it is found in 87% of patients with advanced cervical cancers (Klaes et al. 1999). The frequency of integration also varies by HPV type, with HPV18 exhibiting a significantly higher rate (100%) than HPV16 (76%) (Burk et al. 2017). This distinction is clinically important in cervical cancer development.

Studies have shown that integration breakpoints in cervical cancer and intraepithelial neoplasia frequently occur in the E2 gene, with E2 disruption commonly observed (Klaes et al. 1999, Tsakogiannis et al. 2015). E2 binds to the viral promoter and suppresses the transcription of E6 and E7 (Goodwin and DiMaio 2000). These two oncoproteins play key roles in oncogenesis: E6 promotes the degradation of p53, inhibiting apoptosis, while E7 inactivates pRB, thereby promoting cell cycle progression (Krump and You 2018, Xiao et al. 2025, Zhang et al. 2025). Thus, disruption of E2 leads to unchecked activation of E6 and E7, driving cell immortalization and tumorigenesis. Beyond transcriptional control, E2 also recruits E1 to the replication origin, making it essential for efficient viral replication. In addition, E2 associates with the host mitotic apparatus to ensure stable episomal inheritance and maintenance of viral latency. However, once the HPV genome integrates into the host chromosome, E2 typically loses function. Even if integration of an E2-deficient genome occurs by chance, such cells may be positively selected through E6 and E7 activation (Fig. 5).

More recent comprehensive genome analyses using next-generation sequencing have shown that integration breakpoints are not strictly confined to the E2 region but can occur throughout the HPV genome (Hu et al. 2015, Burk et al. 2017). In some cells where E2 remains intact, E6 and E7 expressions may still be upregulated through alternative mechanisms. These include epigenetic silencing of E2 via methylation at its binding sites, or the formation of fusion transcripts at non-E2 breakpoints that either promote E6/E7 expression or suppress E2 activity—ultimately enhancing E6 and E7 expression (Zhang et al. 2025).

### KSHV and Kaposi’s sarcoma

**Figure 6.**
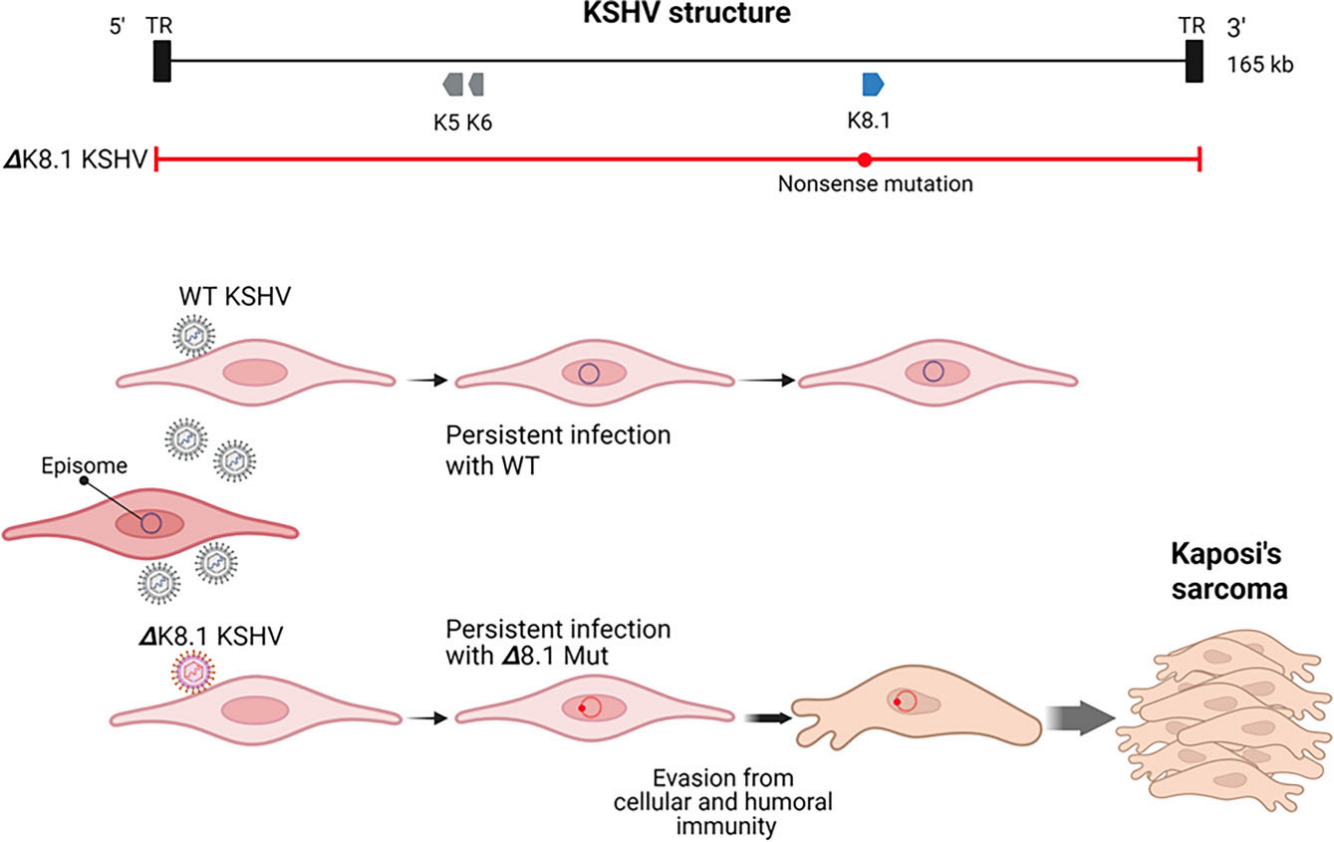
Kaposi’s sarcoma-associated herpesvirus (KSHV) and Kaposi’s sarcoma. Loss of K8.1, a highly immunogenic viral glycoprotein, facilitates evasion from both cellular and humoral immune responses. Created in https://BioRender.com.

KSHV, a large DNA virus of the Orthoherpesviridae family, is associated with several malignancies, including Kaposi’s sarcoma and primary effusion lymphoma (PEL), particularly in immunocompromised individuals (Alaggio et al. 2022, Campo et al. 2022, Damania and Cesarman 2022). The virus primarily targets B cells and vascular endothelial cells, leading to PEL and Kaposi’s sarcoma, respectively (Dittmer and Damania 2016). After infection, KSHV persists in the host cell nucleus in latent form as an episome (Fig. 6).

Viruses harboring functional defects in the K8.1 gene—which encodes a structural glycoprotein—have been detected at relatively high frequency in Kaposi’s sarcoma lesions (8 of 29 cases, 28%) (Santiago et al. 2022). These defects are not large intragenic deletions, as often observed in EBV (another member of the Orthoherpesviridae family) but, rather point mutations that introduce stop codons or deletions affecting transcription start sites. Notably, these inactivating mutations in K8.1 are found exclusively in viral genomes isolated from tumor tissue, while K8.1 remains intact in oral samples from the same patients (Santiago et al. 2021). K8.1 is a glycoprotein that plays a key role in viral infectivity and egress, and deletion of the K8.1 gene significantly impairs these functions—rendering the virus replication-defective. Additionally, K8.1 protein is highly immunogenic, and loss of K8.1 expression could contribute to immune evasion (Olp et al. 2015). However, in the setting of Kaposi’s sarcoma, which typically occurs under immunosuppressed conditions, the extent to which this contributes to oncogenesis remains uncertain. Cells infected with K8.1-deficient viruses may nevertheless gain a selective advantage in the virus-infected tissue microenvironment, allowing them to persist and expand (Fig. 6).

Although not a gene deletion, amplification of the K5–K6 gene region has also been reported in some cases of Kaposi’s sarcoma (Santiago et al. 2022). K5 and K6 are known to function as immune modulators—K5 as an inhibitor of immune recognition and K6 as a viral chemokine—and their overexpression may contribute to immunosuppression in the tumor milieu.

KSHV, like EBV, encodes at least 17 viral miRNAs; however, few intragenic deletions, including these miRNAs have been reported thus far. While comprehensive genomic analyses have been conducted for EBV across various lymphoma types, the number of tumor samples available from PEL remains limited (Marshall et al. 2024, Moorad et al. 2025). Broader studies may provide deeper insight into the role of KSHV genome defects in tumor development.

### MCPyV and Merkel cell carcinoma

**Figure 7.**
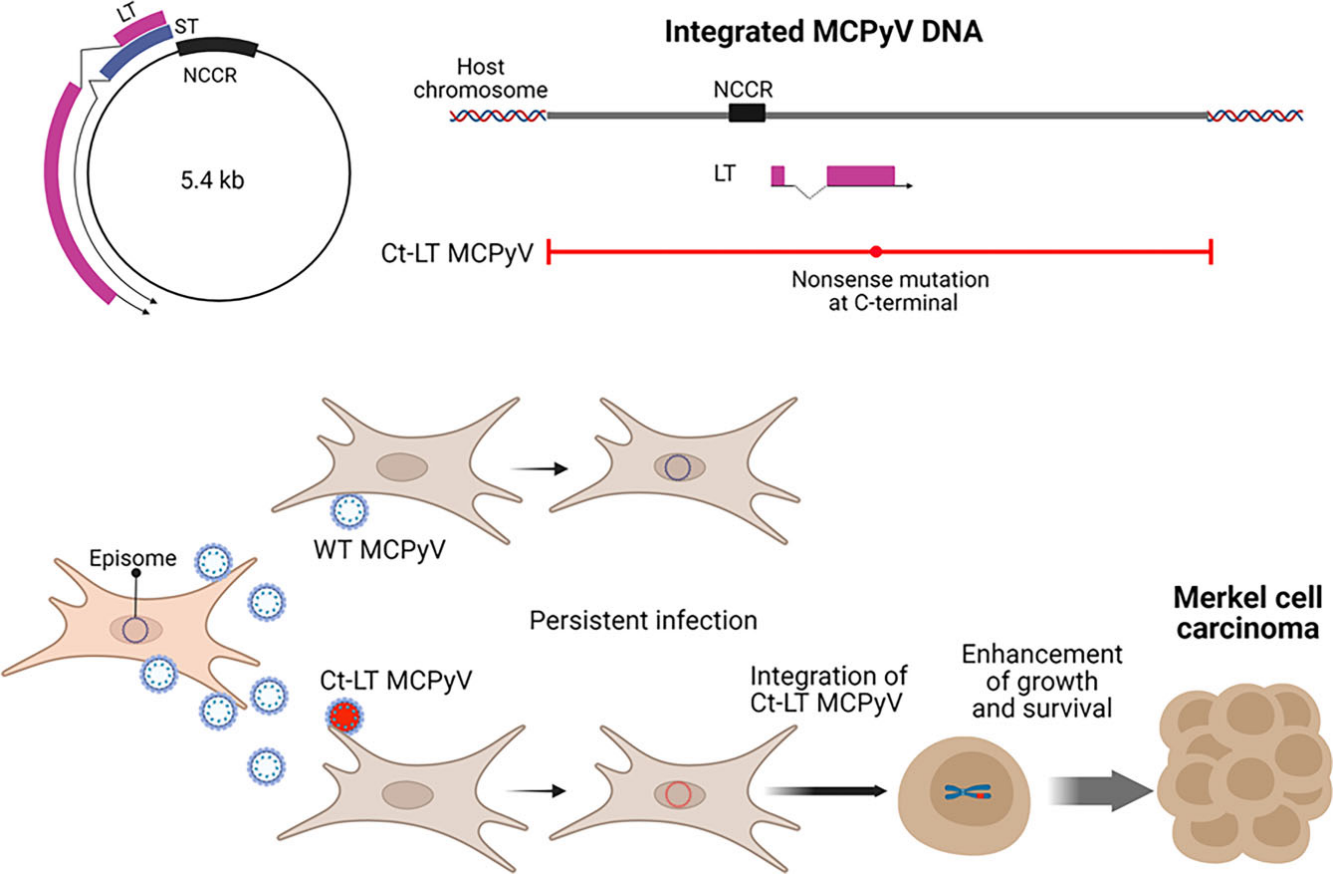
Merkel cell polyomavirus (MCPyV) and Merkel cell carcinoma. C-terminal-truncated large T antigen (Ct-LT), resulting from nonsense mutations prior to genome integration, enhances tumor cell growth and survival. NCCR, noncoding control region; ST, small T antigen. Created in https://BioRender.com.

MCPyV is a small circular double-stranded DNA virus first identified in 2008 from Merkel cell carcinoma tissues using digital transcriptome subtraction (Feng et al. 2008). It is now believed that MCPyV infects skin fibroblasts, rather than Merkel cells as previously thought (Harms et al. 2018). After infection, the virus remains latent in the nucleus in episomal form (Fig. 7). MCPyV is detected in approximately 80% of Merkel cell carcinomas, and all MCPyV-positive cases exhibit clonal integration of the viral genome into host chromosomes (Moore and Chang 2010, Harms et al. 2018). This integration is thought to occur via host DNA damage repair pathways, particularly through non-homologous end joining or microhomology-mediated break-induced replication (Czech-Sioli et al. 2020, Vojtechova and Tachezy 2025). Both host and viral breakpoints appear to be random.

In Merkel cell carcinoma, truncation of the integrated viral LT gene at its C-terminal region is almost universally observed (Harms et al. 2018). LT is a multifunctional protein involved in both the viral life cycle and tumorigenesis. The truncated LT lacks the helicase domain necessary for viral replication, rendering the virus replication-defective. However, its pRB-binding domain remains intact, allowing it to interfere with host cell cycle control and promote oncogenesis. As a result, because of both immune evasion and enhanced proliferative capacity, mutant viruses harboring defective LT are positively selected and enriched as tumorigenic clones (Fig. 7). Thus, MCPyV integration and expression of defective LT are considered central mechanisms in the development of Merkel cell carcinoma (Moore and Chang 2010).

Most LT truncations are caused by point mutations that introduce premature stop codons and are thought to occur prior to genome integration (Czech-Sioli et al. 2020, Starrett et al. 2020). These mutations likely arise from viral replication errors or host DNA repair processes, rather than from UV radiation. Notably, virus-negative Merkel cell carcinomas show a high frequency of UV signature mutations, indicating UV exposure as a primary oncogenic driver in those cases. By contrast, MCPyV-positive Merkel cell carcinomas exhibit fewer UV-associated and host gene mutations, suggesting that MCPyV with defective LT serves as the principal oncogenic driver in these tumors (Harms et al. 2018).

## Clinical implications and future perspectives

Defective viral genomes hold significant potential as biomarkers for early diagnosis. These genetic alterations are rarely found in normal, infected cells but are commonly observed in virus-associated cancers. For instance, truncated LT is almost universally present in MCPyV-positive Merkel cell carcinoma (Moore and Chang 2010). Similarly, deletions of BART miRNAs or EBNA3B in EBV (Venturini et al. 2021, Khine et al. 2025), as well as integration-associated E2 disruptions in HPV (Burk et al. 2017), are strongly linked to cell transformation and persistent infection. A key distinction lies in the fact that tumor-associated viruses often harbor defective genomes, whereas non-oncogenic viruses typically maintain full-length genomes. This virological contrast enables the selective detection of defective viral genomes in clinical specimens. Notably, circulating tumor-derived viral DNA carrying these defects can be identified in blood, offering a non-invasive strategy for diagnosis and disease monitoring. This is especially valuable in liquid biopsy applications, including early cancer detection and minimal residual disease assessment (Li et al. 2020, Vojtechova and Tachezy 2025).

In addition, defective viral mutants are gaining attention as prognostic indicators. For example, HBV PreS/S mutations are associated with increased risk of liver cirrhosis and hepatocellular carcinoma (Liang et al. 2021), and K8.1-deficient KSHV may contribute to the progression of Kaposi’s sarcoma (Olp et al. 2015). The presence of these mutants opens up opportunities to stratify virus-associated tumors based on molecular features such as immune responsiveness, viral antigenicity, or the degree of tumor cell dependency on the virus.

From a therapeutic standpoint, virus-associated tumors harboring integrated genomes with defective replication capacity are typically resistant to standard antiviral therapies. This is because most defective viral genomes have lost the ability—and the necessity—to replicate, while conventional antiviral agents are designed to target viral replication machinery. However, viral oncogenes that remain active in tumors—such as HBx, HBZ, E6/E7, and MCPyV LT—can serve as targets for small-molecule inhibitors, RNA interference, T-cell–based therapies, and therapeutic vaccines (Mesri et al. 2014, Chokwassanasakulkit and McMillan 2024, Wong and Yee 2024, Xiao et al. 2025).

Furthermore, defects and mutations can alter viral immunogenicity, influencing the efficacy of immune checkpoint inhibitors. In Merkel cell carcinoma and EBV-positive lymphomas, specific defective mutations can shift antigenicity and potentially affect tumor sensitivity to PD-1/PD-L1 blockade. Notably, the effectiveness of avelumab and pembrolizumab has been reported in Merkel cell carcinoma (Becker et al. 2017, Harms et al. 2018). A deeper understanding of viral genetic abnormalities will directly aid in patient stratification and contribute to the refinement of immunotherapeutic strategies.

## Closing remarks

Defective viral genomes are not merely byproducts of viral replication but play active roles in virus-driven oncogenesis. Their selective retention across diverse oncoviruses highlights common strategies for immune evasion, persistence, and cell transformation. Recognizing these genomes as tumor-promoting agents opens new avenues for novel approaches in diagnosis, prognosis, and targeted therapy for virus-associated cancers.
